# Controlling CO_2_ emissions for each area in a region: the case of Japan

**DOI:** 10.1186/s13021-019-0135-7

**Published:** 2019-12-27

**Authors:** Tetsuya Tamaki, Wataru Nozawa, Shunsuke Managi

**Affiliations:** 1 Faculty of Engineering and Design, Kagawa University, 2217-20 Hayashi-cho, Takamatsu, Kagawa 7610396 Japan; 2 Department of Economics, Fukuoka University, 8-19-1 Nanakuma, Jonan, Fukuoka, 8140180 Japan; 3 Department of Urban and Environmental Engineering, School of Engineering, Urban Institute, Kyushu University, 744 Motooka, Nishiku, Fukuoka, 8190395 Japan

**Keywords:** Climate policy, The RICE model, 2.5° limit, $\text {CO}_2$ emission reduction

## Abstract

**Background:**

Global warming is the most serious problem we face today. Each country is expected to ensure international cooperation toward minimizing risk. To evaluate the countermeasures, many researchers have developed integrated assessment models (IAMs). Then, how can each country achieve its emission quota? This study proposes models that analyze the economic impact of global warming in a region based on the results obtained by the global model. By using these suggested models, we perform a comparative analysis on three policy cases: a different regulations case, a unified regulation case, and an output redistribution case.

**Results:**

We analyzed Japan as one of the case studies and found that more developed areas should implement stricter regulations in all scenarios. In addition, the case of applying different regulations by area (in a region) is not always preferable to using unified regulations in the region. Alternatively, the output gap between the output redistribution case and the different regulations case is much higher than the gap between the unified regulation case and the different regulations case. In all scenarios, the present values of the output of the output redistribution case are also higher than the other cases.

**Conclusions:**

The different regulations case and the unified regulation case are based on the model without capital transfer between areas, whereas the output redistribution case is based on the model with free capital transfer between areas. Although both models are extreme situations, the regions close to the without capital transfer situation possibly have an incentive to use the different regulations policy, depending on the emission target. The regions close to the situation with free capital transfer would probably prefer unified regulation.

## Background

### Introduction

Global warming is one of the most serious issues in the world, and the nations of the world need to stand together globally to tackle the problem. In 2018, the Intergovernmental Panel on Climate Change (IPCC) reported that the average global temperature is already more than 1° higher than the temperature in the pre-industrial era and will be 1.5° higher between 2030 and 2052 [12].

The 2015 United Nations Climate Change Conference (COP21) was held in Paris and it was agreed to set a 2° limit on average global temperature rise (compared with that of the pre-industrial era). Each country submitted its contribution plan. For example, Japan planned to reduce 26% of greenhouse gases (GHGs) by 2030 (relative to 2013). Many other countries also aimed to reach their GHG-reduction goals; however, the total reduction of GHGs would not be sufficient to avoid exceeding the 2° limit [31]. Although the governments could adopt a robust set of guidelines for implementing the 2015 Paris Agreement in COP24 in 2018, they were unable to “welcome” the IPCC report proposing a temperature rise limit of 1.5° [11]. The global mean surface temperature is one of the best measurements because it has a wider coverage, lower uncertainty in future projections, and richer historical observation records than many other instruments [17]. However, several factors influence the stricter agreements on global warming. Global warming has important features such as being international, long-term, uncertain, and irreversible. Wagner and Weitzman [43] suggests that implementing countermeasures for global warming can be difficult. Furthermore, it might induce misunderstanding about the scientific consensus on global warming. For example, most climate scientists have concluded that climate change results from human activities [23], but many other people doubt that global warming is caused by humans [21]. Van der Linden et al. [41] stated that the level of perceived scientific agreement influences the level of support for public actions. Global economic inequality is also an obstacle to global warning countermeasures. In COP24, although all countries finally reached an agreement, it included either ambiguous expressions or inconsistent provisions [11].

However, the difficulty in coming to a consensus on global warming countermeasures does not mean that action will not be taken. We need to figure out not only a framework but also the impact of global temperature rise. We also need to estimate the cost for each country to achieve the most suitable scenario. This study divides the burden of GHG reduction in a region. Although temperature rise limits, such as by 2° and 1.5°, are determined globally, it remains uncertain how each country should reduce its GHG emissions to avoid exceeding the limit. Models such as the regional integrated climate-economy (RICE) model developed by Nordhaus can calculate the optimal emission amounts for several regions in the world [29]; however, these models are tools for analyzing global impact and effects. It is necessary to clarify each country’s response to global warming while maintaining these models’ results. Moreover, economic conditions vary regionally within each country. It is important to consider each country’s strategy for tackling the global warming problem together.

The rest of this paper is organized as follows. “Literature review” subsection reviews the current literature and clarifies the scope of this research. “Methods” section shows the RICE model and our models, and “Results and discussion” section discusses the results. Finally, conclusions and suggestions for future study are summarized in “Conclusions” section.

### Literature review

The economic impact of global warming has been widely analyzed since the 1990s. Some estimate the integrated global impact by IAMs like the dynamic integrated climate-economy (DICE) model [26]; others study the local impact, such as natural disaster risks [8, 42] and human health risks [6, 18]. Although both are indispensable for examining the countermeasures of global warning, the former is more important in making comprehensive decisions. The two get lumped together as IAMs, but there are different types of IAMs [4, 37]: general equilibrium models [1], simulation models [7, 9], and optimization models [5, 26]. The DICE model is one of the most famous models, and many researchers have discussed IAMs using the DICE model. For example, some studies introduce new models based on the DICE model [3, 34, 35, 38, 40]. However, the DICE model has also been criticized. Kaufmann [16] stated that the DICE model underestimates the impact of global warming due to unsupported assumptions and simple extrapolations. Hu et al. [10] pointed out that the DICE model is sensitive to the means and covariance of the parameters. However, this criticism does not stop researchers from using the DICE model. Nordhaus [25] stated that “one of the major shortcomings of IAMs is that their structure makes it extremely difficult to use standard econometric techniques to assess their reliability.” He examined both the first published model [26] as well as the latest version [27] of the DICE model. Although the abovementioned features of global warming might affect estimation accuracies, we still need to address these issues. Integrated assessment models have the potential to estimate the comprehensive impact of global warming. Additionally, it is important to build the models because they are helpful in formulating the countermeasures and policies.

In the case of global warming, each government needs the information not only about the global impact but also about the impact on their own country or region. The regional impact of global warming is also estimated by many researchers, and it varies depending on the region. Each country or region has unique geographic, social, economic, and environmental features. For example, agriculture, which is directly affected by global warming, is the subject of many research studies [24, 33]. Kurukulasuriya et al. [20] focused on African agriculture and stated that the crops relied solely on rainfall and the livestock are vulnerable but suggested that promoting irrigation could help alleviate the possible effects of climate change. Kunimitsu [19] estimated the economic impact of damage to rice production in Japan. Lioubimtseva and Henebry [22] focused on Central Asia and analyzed the more comprehensive impact of climate change by assessing the vulnerability of the region to various global warming risks, e.g., food safety, water stress, and public health.

Policies on global warming are implemented by countries and regions. A typical example is the European Union’s emission trading scheme (EUETS), launched in 2005. It remains the largest GHG-ETS in the world. By 2020, the EU wants to cut down GHG emissions by 20% below the 1990 level. According to an OECD report, the EUETS has not hurt revenues, profits, or employment at any firms in the EU [30]. The ETS has also been introduced to other countries such as Korea [32], New Zealand [15], and China [14]. In Japan, it was adopted in Tokyo in 2010, and emissions from facilities under the program have been significantly reduced [39].^1^ However, as mentioned in "Background" section, the total emission reductions submitted by all countries is still insufficient to meet the 2° or 1.5° target. Although this reduction is important, it is necessary to link it with the final objective. We need to figure out more feasible actions by dividing the global goal into individual country or regional goals based on the result obtained by the global model.

## Methods

This study proposes the method based on the RICE model, which is derived from the DICE model and can estimate the risks of several regions. Both the models basically have the same structure. One of the key differences lies in the objective function. The RICE model maximizes the sum of utilities of the regions, whereas the DICE model uses one utility function for the entire world. Concomitantly, the regional productivities are defined in the RICE model.

1 $$\begin{aligned} W= & {} \sum _{i}^{N}\sum _{t}^{T_{max}}(1+\rho )^{-t+1}U_{i,t}\left( L_{i,t}, c_{i,t}\right) \end{aligned}$$

2 $$\begin{aligned} U_{i,t}\left( L_{i,t}, c_{i,t}\right)= \,& {} L_{i,t}\frac{[c_{i,t}]^{1-\alpha }}{1-\alpha } \end{aligned}$$

Equation (1) is the objective function in the RICE model. The subscripts *i*, $i=1,\ldots , N$, and *t*, $t=1, \ldots , T_{max}$, represent the region and the period, respectively. $\rho$ is the time preference rate, and *U* is the utility, which is defined by the population *L* and the per capita consumption *c*. In Eq. (2), $\alpha$ is the elasticity of intertemporal substitution. The production function is shown as follows:

3 $$\begin{aligned} Q_{i,t}=A_{i,t}K_{i,t}^{\gamma }L_{i,t}^{1-\gamma }[1-\Lambda _{i,t}][1-\Omega _{i,t}] \end{aligned}$$

where *A* and *K* denote the total factor productivity (TFP) and the capital, respectively. $\gamma$ is the elasticity of output with respect to capital. $\Lambda$ and $\Omega$ indicate the abatement cost ratio and the climate damage ratio, respectively. It is assumed that the abatement cost ratio is determined by the emission control rate $\mu (0\ge \mu \ge 1)$, which is a variable deciding the $\text {CO}_2$ emission level. $\mu =0$ means that $\text {CO}_2$ emission is not regulated at all and the abatement cost becomes zero. Conversely, $\mu =1$ means that $\text {CO}_2$ emission is completely regulated and the abatement cost becomes considerably higher. However, the climate damage ratio is assumed to be the function of the increase in atmospheric temperature *T*. These functions are given by

4 $$\begin{aligned} \Lambda _{i,t}=\, & {} \theta _{1,t} \mu _{i,t}^{\theta _2} \end{aligned}$$

5 $$\begin{aligned} \Omega _{i,t}=\, & {} \phi _{1,i} T_{t}^2+\phi _{2,i} T_{t}+\phi _{3,i} \end{aligned}$$

where $\theta _{1,t}$, $\theta _{2}$, $\phi _{1,i}$, $\phi _{2,i}$, and $\phi _{3,i}$ are the parameters. Moreover, the gross output *Q* is divided into consumption $C (C=c\cdot L)$ and investment *I*:

6 $$\begin{aligned} Q_{i,t}=C_{i,t}+I_{i,t}. \end{aligned}$$

The capital *K* is expressed by

7 $$\begin{aligned} K_{i,t}=I_{i,t}+(1-\delta )K_{i,t-1}, \end{aligned}$$

where $\delta$ is the capital depreciation rate. The $\text {CO}_{2}$ emission is assumed to be proportional to the net output, or,

8 $$\begin{aligned} E_{i,t}=\sigma _{i,t} [1-\mu _{i,t}]A_{i,t}K_{i,t}^{\gamma }L_{i,t}^{1-\gamma } +\eta _{i,t}. \end{aligned}$$

where $\sigma _{i,t}$ is the level of carbon intensity; $\eta _{i,t}$ is the amount of $\text {CO}_2$ emission from land use, given as an exogenous variable. The emission gas circulates in the atmosphere and the ocean, thus the concentration of $\text {CO}_2$ for the period is decided by the carbon cycle model in the RICE model [28]. Moreover, the concentration of $\text {CO}_2$ increases radiative intensity, indicating an average temperature rise. As we can see from Eq. (5), rising temperatures lead to a decrease in gross output. Consequently, we determine the economic activity level and the emission control rate considering the balance of the economy and the environment.

**Fig. 1:**
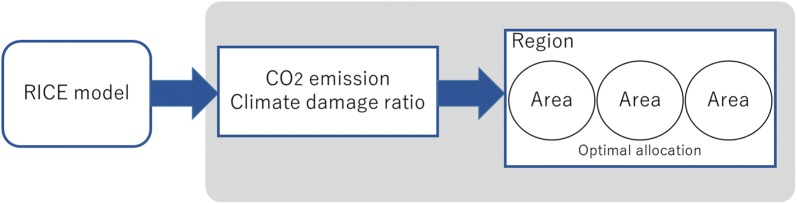
The RICE model and this model

Our model estimates the optimal economic activity by each area based on the RICE model. Here, we supposed a two-stage approach. The reasons are as follows. First, our model can be calculated without the RICE model if we can know the target emission of the region and the anticipated climate change damage. Our model can easily be applied to another models. Second, the RICE model may lead to infeasible optimization problems if we modify the model to fit a more complex analysis. Nordhaus and Sztorc [28] mentioned that the RICE models are prone to errors in the software and structure when modifications are made to the models. Finally, we were concerned about evaluating different regional levels in the same model. Schumacher [36] insisted that different levels of aggregation can potentially lead to different results. The RICE model divides the world into several regions. It has already identified a single country or a region where multiple countries are combined; hence, subdividing areas of a country should be avoided.

Figure 1 describes the relationship between the RICE model and this study’s model. The shaded area indicates the focus of this study. Using the RICE model, we can analyze our best action for climate change and estimate how many degrees the temperature will rise and how much we should regulate the $\text {CO}_2$ emissions. This study attempts to analyze the optimal strategy in the subdivided areas using the result of the RICE model. If we know how much we should limit the $\text {CO}_2$ emission from a region during each period, each area in the region will be given the optimal allocation of $\text {CO}_2$ emissions. The model can be formulated as follows:

9 $$\text {max} \quad W= \sum _{j}^M\sum _{t}^{T_{max}}(1+\rho )^{-t+1}U_{j,t}\left( C_{j,t}\right)$$

10 $${\text {s.t.}} C_{j,t}= Q_{j,t}-I_{j,t}$$

11 $$Q_{j,t}= A_{j,t}K_{j,t}^{\gamma }L_{j,t}^{1-\gamma }[1-\Lambda _{j,t}][1-\Omega _{0,t}]$$

12 $$K_{j,t+1}= \left( 1-\delta \right) K_{j,t}+I_{j,t}$$

13 $$\Lambda _{j,t}= \theta _{j,t}^{1} \mu _{j,t}^{\theta ^{2}}$$

14 $$E_{j,t}= \sigma _{j,t} [1-\mu _{j,t}]A_{j,t}K_{j,t}^{\gamma }L_{j,t}^{1-\gamma }$$

15 $$E_{0,t}\ge \sum _j^M E_{j,t}.$$

This model focuses on the areas within a region in the RICE model and is based on Eqs. (1)–(8). The region set is denoted by ${\mathbb {M}}=\left\{ 1, \ldots , M\right\}$, and the subscript $j \in {\mathbb {M}}$ represents the areas in the region *j*. It assumes that the economic activity in the area *j* is similar to that in the region in the RICE model (Eqs. 10–14). Note that the climate damage ratio, $\Omega _{0,t}$, is used as an exogenous variable, whereas it is an endogenous variable in the RICE model. Additionally, the upper bound of the total $\text {CO}_2$ emission, $E_{0,t}$, is given as a constraint in this model (Eq. 15). These assumptions are derived from the optimal paths of temperature increase and $\text {CO}_2$ emission increase provided by the RICE model. When the increased level of atmosphere temperature is determined, the climate damage ratios in all areas are also determined based on Eq. (5). This model selects one region in the RICE model, so the areas within the region have to limit their $\text {CO}_2$ emissions below the optimal emission level estimated by the RICE model. Hence, the total emission is equal to the emission in the region when maximizing the objective function. Then the increased level of atmosphere temperature is the same as the result of the RICE model. Note that the suggesting model is the optimization model given the constraints on the emission amount and the climate damage ratio (or the temperature rising level). Although it can not estimate the best strategy in consideration of the other regions except in special cases, it is possible to analyze the optimal behavior of the region under the several situations. This study analyzes three cases based on the model. These cases are introduced in "Emission control strategies: three cases" section.

**Fig. 2:**
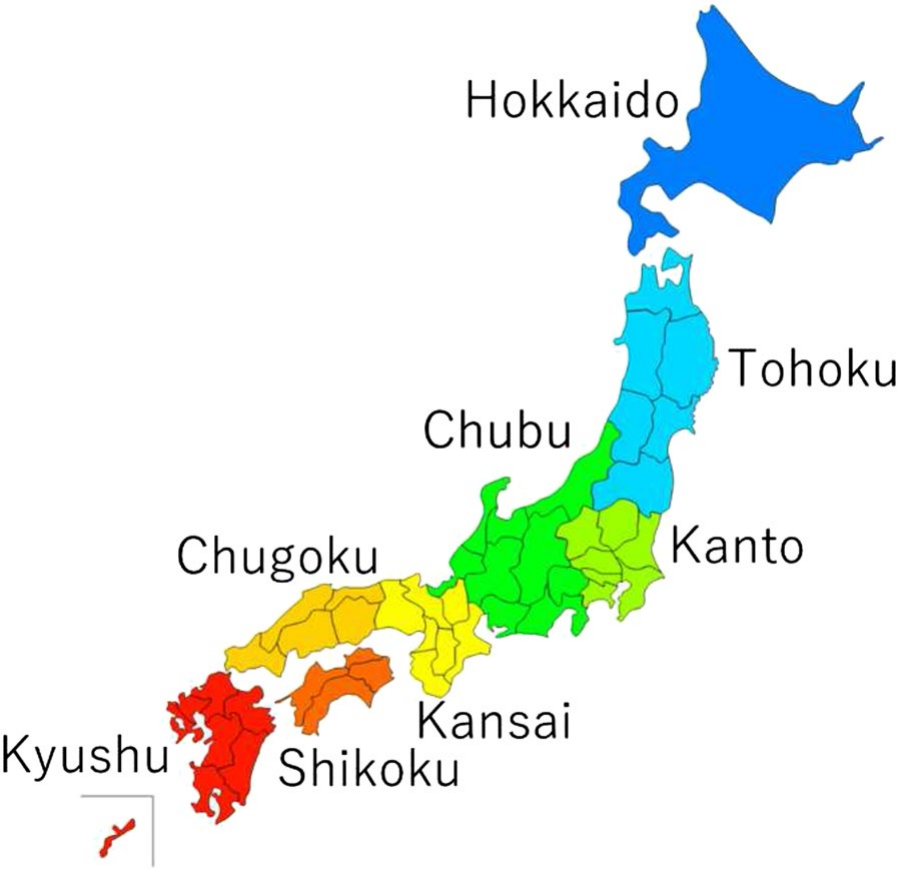
The subdivided areas in Japan

The case of Japan is demonstrated by using this model in the following section. In the model, Japan is divided into eight areas (Hokkaido, Tohoku, Kanto, Chubu, Kinki, Chugoku, Shikoku, and Kyushu (see Fig. 2). The time step and the periods are assumed to be 5 years and 60 (300 years), respectively, according to Nordhaus [27]. The main parameters are listed in Table 1. The initial values and the exogenous variables used in this analysis are shown in Appendix A.

The population data are based on the statistics data from the cabinet office, Government of Japan (CAO) and the National Institute of Population and Social Security Research (IPSS). IPSS estimates the population for each prefecture up to 30 years in the future (2045). According to IPSS [13], the population in Japan will be approximately 100 million in 2045 and will decrease in the future, but population projections often involve uncertainty. Hence, CAO [2] suggests nine scenarios that predict population growth. In this analysis, we use one of the scenarios that converges to 100 million in the future. We assumed that the population will converge to the estimated 2045 population size in the future.

As for the damage functions of each area, we assumed the same damage level in all areas. It would be preferable to use different damage functions for each area, but the estimation is complicated and troublesome. In particular, the damage function used in the RICE model is a rough estimate that uses temperature as a unique variable. Therefore, we decided that it would better to reuse the damage function of Japan as a unified function.

**Table 1:** Parameters

Parameter		Value
$\rho$	The time preference rate per 5 years	0.075
$\alpha$	The elasticity of intertemporal substitution	1.45
$\gamma$	The elasticity of output	0.3
$\theta ^2$	Adjustable parameter	2.6
$\delta$	The capital depreciation rate	0.1

## Results and discussion

### Three climate change scenarios

**Fig. 3:**
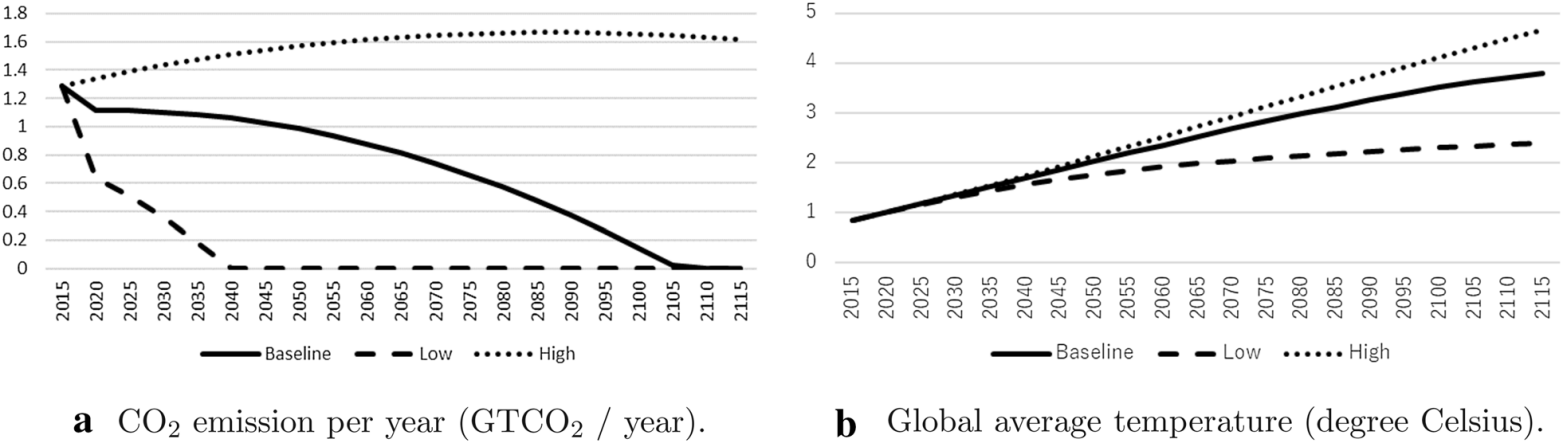
Three scenarios

We interpreted the results of three different scenarios in this study. The scenarios were based on the exogenous variables that were estimated by the RICE model. The climate damage ratio, $\Omega _{0,t}$, and the upper bound of the total $\text {CO}_2$ emission, $E_{0,t}$. *Baseline* used the results from the optimization in the RICE model. The DICE and the RICE model show that the average temperature will rise more than 3° in a hundred years if we promote the most effective anti-global warming policies that consider a balance between the abatement cost and the benefit of reduced climate change. The second scenario was labeled *High*. This is the scenario under the condition that the radiative intensity becomes more than $8.5\,\text {W/m}^2$. This scenario corresponds to the situation where we conduct economic activity without worrying about GHG emissions. On the other hand, the scenario labeled *Low* corresponds to the situation where we conduct economic activity with strict emission regulations. Although the IPCC considers the RCP2.6 scenario to be a low emission scenario, the RICE model works well because it is unrealizable [27]. Therefore, this study uses the 2.5° temperature rise limitation as a low emission scenario. Figure 3 shows the exogenous variables estimated by the RICE model. As shown in the difficulty of RCP2.6 scenario analysis by the RICE model, the Low scenario is also one of the very strict targets in the RICE model. Although the scenario may be unrealistic, we calculate the scenario for comparison with the other scenarios.

### Emission control strategies: three cases

In this study, we analyzed the gap in the impact by comparing different ways of emission control regulation. One is the case where different regulations are used for each area, the other is the case where a unified regulation is used in all areas. In the case of different regulations for each area, we used the model introduced in "Methods" section; Eqs. (9)–(15). This model can decide the optimal control rates by each area under the constraints of each scenario. Analyzing the case for a unified regulation in all areas, we add the following constrains to the model constructed by Eqs. (9)–(15),

16 $$\begin{aligned} \mu _{i,t}=\mu _{j,t}, \forall i,j \in {\mathbb {M}}. \end{aligned}$$

Equation (16) represents the situation where the emission control level is the same in all areas.

In addition, we considered the case of output redistribution. The previous two cases did not assume the exports and imports between areas. These cases describe the model in which the output produced from a certain area is consumed only in that area and is invested only as capital for that area. The third case considers the situation where output can be freely transferred between areas. For simplicity’s sake, it is assumed that there are no transfer costs such as transportation costs. The model is

17 $$\text {max} \ \, W= \sum _{j}^M\sum _{t}^{T_{max}}(1+\rho )^{-t+1}U_{j,t}\left( C_{j,t}\right)$$

18 $$\text {s.t.} \quad C_{j,t}= Y_{j,t}-I_{j,t}$$

19 $$Q_{j,t}= A_{j,t}K_{j,t}^{\gamma }L_{j,t}^{1-\gamma }[1-\Lambda _{j,t}][1-\Omega _{0,t}]$$

20 $$\sum _j^M Y_{j,t}= \sum _j^M Q_{j,t}$$

21 $$K_{j,t+1}= \left( 1-\delta \right) K_{j,t}+I_{j,t}$$

22 $$\Lambda _{j,t}= \theta _{j,t}^{1} \mu _{j,t}^{\theta ^{2}}$$

23 $$E_{j,t}= \sigma _{j,t} [1-\mu _{j,t}]A_{j,t}K_{j,t}^{\gamma }L_{j,t}^{1-\gamma }$$

24 $$E_{0,t}\ge \sum _j^M E_{j,t}.$$

This model is similar to the two previous models, but the decisive difference is expressed in Eq. (20). This equation describes the sum of the outputs produced by each area as being equal to the sum of the outputs redistributed to each area. Although this model does not include the constraint that the emission control level is the same in all areas the way Eq. (16) does, the result becomes the same emission control level if the climate damage ratio is same in all areas (see Appendix B).

The results of these models are discussed in the following sections.

### Emission allocation

**Fig. 4:**
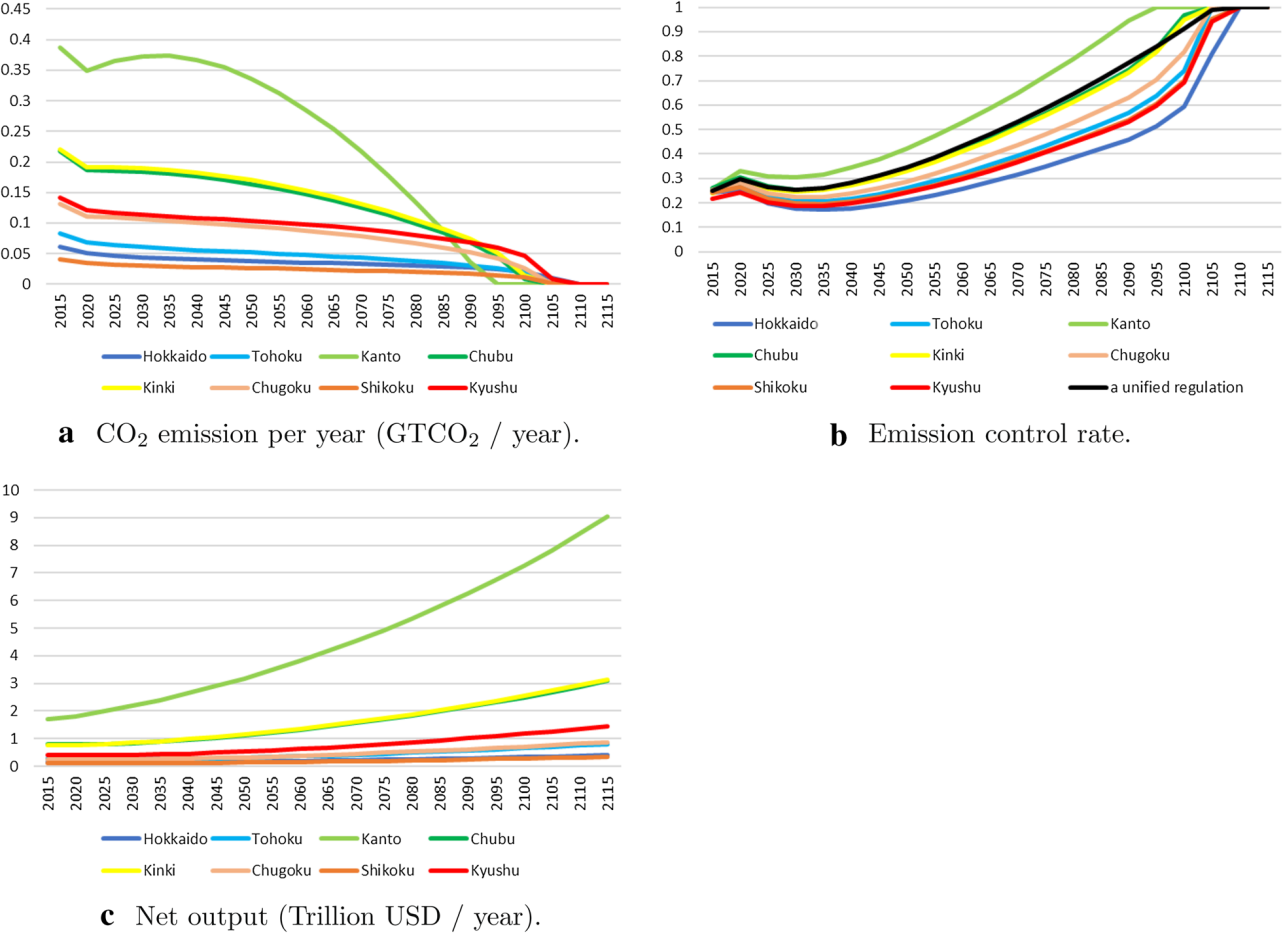
*Baseline* scenario

Figure 4 describes the $\text {CO}_2$ emissions from each area within a region, the emission control rates, and the net output of the *Baseline* scenario. The Kanto area, including Tokyo, is the largest economic area in Japan, followed by Chubu and Kinki. The Kanto area generates large amounts of $\text {CO}_2$ compared with other areas during this period, and the results show that the Kanto area should completely regulate $\text {CO}_2$ emissions until 2095. However, Chubu and Kinki should stop releasing $\text {CO}_2$ until 2105. All of the areas have to completely stop emissions until 2110, which means that it is desirable to adopt different regulations by each area to realize the most preferable economic activities, and the larger economic areas should set stricter regulations. The gaps in regulation levels among the different areas stem from the difference in regulatory impact. The regulations in relatively developed areas have less impact than those in less developed areas.

The maximum gap in the starting points for completely stopping the emissions is approximately 15 years among the areas. The emission control rate of Hokkaido is approximately 0.5, whereas that of Kanto reaches 1 in 2095. Moreover, the black line in Fig. 4b illustrates the situation where all areas in Japan chose the same control rate, similar to that in the Chubu and Kinki areas (where we made different policies by each area within a region). This means that if Japan adopts the different policy scenario, areas other than Kanto cannot make stricter regulations than those in the unified regulation scenario. However, the results show that these areas need to sharply tighten their regulations in the future. For example, the emission control rate will rise from 0.6 to 1 between 2100 and 2110, whereas it will go from 0.2 to 0.6 between 2015 and 2100 in Hokkaido. In the case of unified regulation, the rate will rise from 0.9 to 1 between 2100 and 2110. Although the abatement cost in the far future tends to be a large burden for low economic areas, the areas can wait before depressing the abatement cost.^2^

The outputs of all areas grow as shown in Fig. 4c.^3^ They are expected to increase by approximately five times in a hundred years in Kanto. Even in Hokkaido, where the increase rate is the lowest, it will increase by nearly three times in a hundred years.

**Fig. 5:**
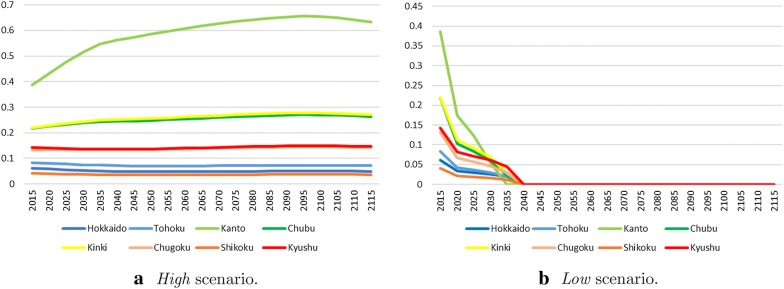
$$\text {CO}_2$$ emission ($$\text {GTCO}_2$$/year)

Figure 5 shows the $\text {CO}_2$ emissions of *High* and *Low* scenarios. In the *High* scenario, we do not regulate the $\text {CO}_2$ emissions. Hence, the emission control is zero between 2020 and 2095. The regulation will start in 2100 because the abatement cost will come down sufficiently, but it will be slack. In contrast, the *Low* scenario needs strict regulation. As evident from Fig. 5b, all areas stop emitting $\text {CO}_2$ after 2040. Both scenarios show that the more developed an area becomes, the stricter its regulation is. However, the gap in complete regulation starting points among the areas becomes smaller as the scenarios become stricter. Early regulations are needed to achieve the strict goal; therefore, the gaps of regulation starting points are smaller as the goal gets stricter.

**Fig. 6:**
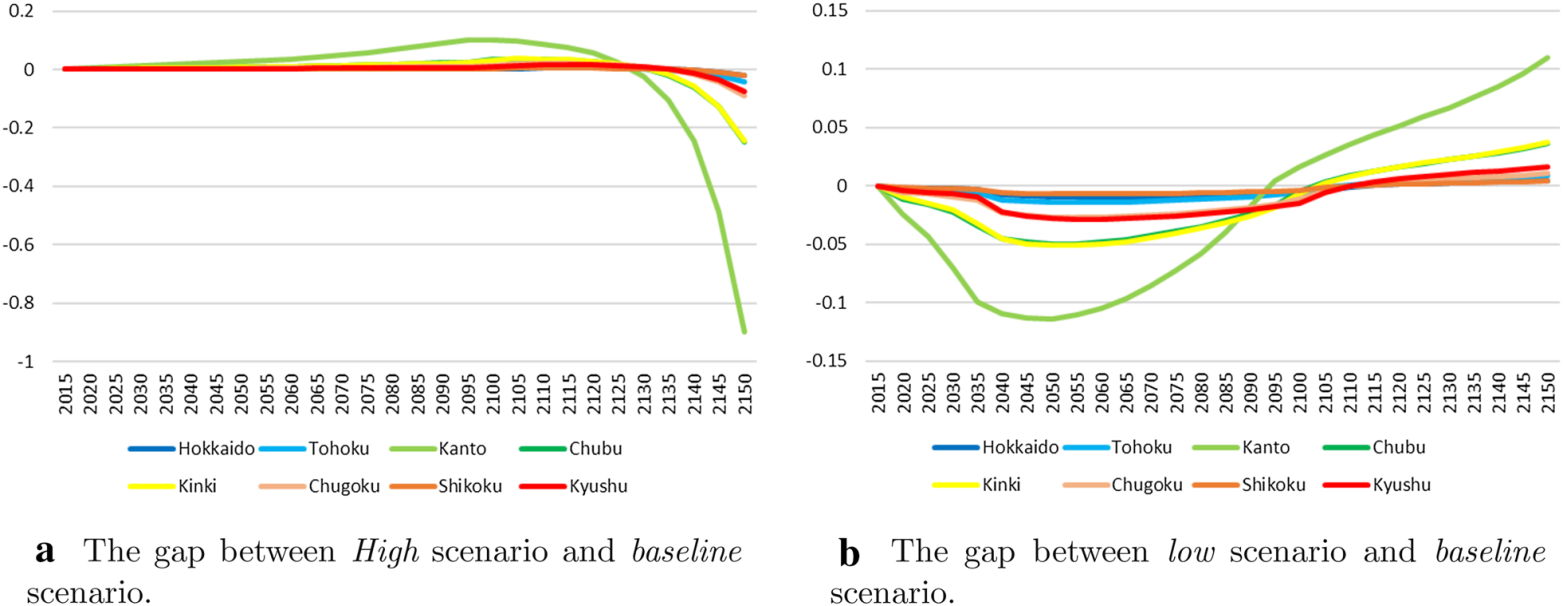
The gap of output (Trillion USD/year)

Adopting *High* and *Low* scenarios has economic impact. Figure 6 compares the output gaps of the *baseline* scenario with these scenarios. In the case of the *High* scenario, there is no $\text {CO}_2$ regulation for a long period of time. This has the potential to increase output in a hundred years, but a serious decline in output is expected after that. The temperature rise will seriously affect future generations. In particular, there will be significant impact on the Kanto area. Compared with the *Baseline*, it will gain up to 74 *billion US*/*year but will lose more than* 1 trillion US/year in 2150 (see Fig. 6a), and the gap will increase. The opposite results are obtained in the *Low* scenario. The output of *Low* is initially below the output of the *baseline*; however, *Low* is expected to have larger outputs than *Baseline* by around 2100.

### The impact of different regulations in a region

As mentioned in "Emission allocation" section, it is the best policy to implement stricter regulations in highly developed areas. In this study, best means the strategy that maximizes the objective function (see Eq. 9). In other words, it maximizes the sum of the utilities of all areas in our model.

**Fig. 7:**
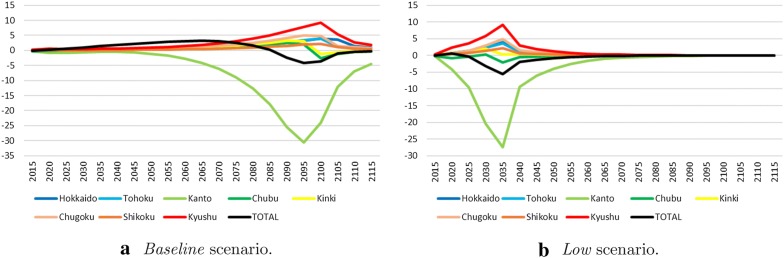
The gap of outputs between different regulations case and unified regulation case (billion USD/year)

Figure 7 shows the gap in outputs between the cases of different regulations for each area and the case of a unified regulation for all areas. The gaps are calculated by subtracting a unified regulation case from different regulation cases in each scenario. By using different regulations in each area, Kanto is most negatively impacted compared to using a unified regulation. The loss is up to approximately 25–30 billion US/year in the *Baseline* and *Low* scenarios. However, Kyushu, Chugoku, and other areas are positively impacted because Kanto takes the burden from these areas. In the *High* scenario, each area has almost no limit on $\text {CO}_2$ emissions in both cases. Therefore, the gap is small compared with the *Baseline* and *Low* scenarios.

The most remarkable point is the total output which is indicated by the black line, which represents Japan. The values are between $+5$ and $-5$ (billion USD/year) in all scenarios and is small in comparison to the gap between scenarios (see Fig. 7), but there is a potential to manage the cost of close to 5 billion USD per year by reviewing our own policies. In addition, the line presents positive values until 2085 and then shows negative values in the *Baseline* scenario. In *Low* scenario, although the values are slightly positive at first, the scenario lacks positive values in the rest of the periods. This indicates that the total output is reduced by using the different regulations of each area. One of the reasons is that the sum of the utilities is used as the objective function. The utility function is convex and monotonically increases with respect to the consumption per capita. Therefore, the objective function will be larger if the consumption gaps among areas become smaller, even though the total consumption is the same. The fact that developed areas bear the $\text {CO}_2$ reduction cost helps the correction of unequal development of domestic economies in Japan. Hence, in spite of the decrease in total output, it is possible to realize high utility compared with the case of universal regulation in *Low*.

From these results, we found that setting optimal regulations for each area does not always increase the total output. As in the case of the *Baseline*, setting regulations for each area has the potential to partly increase the output to the same level of the total emissions. However, there is a possibility of no increase in the output such as in the *Low* scenario. Focusing on the case of the *Baseline* (see Fig. 7a), the gap in the outputs is positive for a relatively long period, whereas it is negative for a short period. Larger total outputs, as well as high total utility, would be achieved compared with those in the unified regulation scenario in Japan. The policy of controlling $\text {CO}_2$ emissions by each area in Japan is one of the most useful countermeasures in that sense. However, it cannot be said that this is effective. In the case of *Low*, the output gap does not become positive, but the total utility has slightly increased. This means that the case of different regulations for each area can only result in less production than in the case of unified regulation in Japan. In such a situation, the different regulation case is not always preferable as suggested by the decrease in total output. Table 2 describes the total outputs from 2015 to 2115 converted into the present values at three different discount rates (1%, 3%, 5%). The different regulations case results in higher outputs than unified regulation case in the *Baseline* scenario, whereas in the *Low* scenario, the opposite result is obtained. In the *High* scenario, the output gap between the two cases is quite small; however, the larger output gap depends on the discount rate in each case.

**Table 2:** Cumulative discounted total output (Trillion USD) [2015–2115]

Discount	Baseline			Low			High		
Rate (%)	D	U	R	D	U	R	D	U	R
1	183.844	183.829	183.952	180.918	180.931	181.005	185.621	185.620	185.682
3	145.065	145.051	145.168	142.563	142.575	142.654	146.422	146.421	146.488
5	116.498	116.485	116.597	114.343	114.354	114.436	117.547	117.548	117.618

*D* different regulations case,* U* unified regulation case,* R* output redistribution case

Selecting the best policy is a matter of deciding which factors are most important. In terms of the present value of the output, in the *Low* scenario, it would be best to choose a unified regulation. In addition, unified regulation produces more output in almost every time period. When distributing the surplus to the other areas (in the unified regulation case), each area will have more output than that in the case of different regulations. Hence, it is possible to produce higher utility than in the different regulation case in all areas. The model proposed in "Methods" section is based on the RICE model, and it does not consider the transfer of capital between areas. Depending on the conditions, the results indicate that the transfer of capital between areas can affect the countermeasures of global warming more than changing emission regulations by area.

**Fig. 8:**
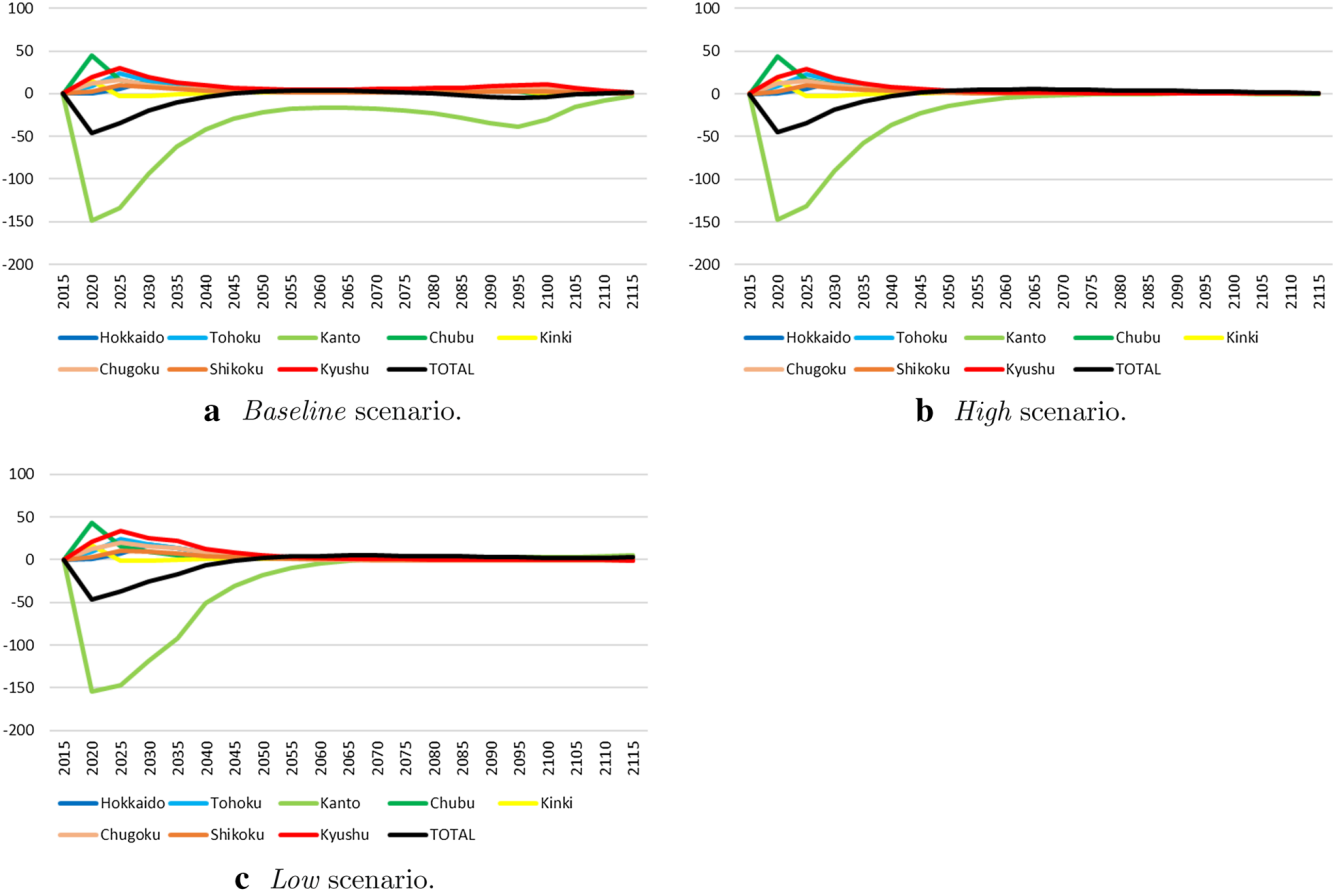
The output gap between the different regulations case and the output redistribution case (billion USD/year)

The case where each area can export and import the other areas’ output was analyzed using the model of Eqs. (17)–(24). As mentioned in "Emission control strategies: three cases" section, this case assumes no transfer costs between areas, or it means that the transfer costs are infinite. Although both assumptions are extreme cases, a realistic situation lies between them, so it makes sense to consider the cases. Figure 8 shows the subtraction of the output redistribution case from the different regulations case. The case of output redistribution would have the potential to implement higher outputs than would the idea of surplus distribution, mentioned in the previous paragraph because it is based on the optimization model of Eqs. (17)–(24). In fact, as shown in Fig. 8, the gap with the output redistribution case is much higher than with the unified regulation case (Fig. 7). The values are up to approximately 50 billion USD/year. In all three scenarios, the present values of the output in the case of output redistribution are also higher than in the other cases (Table 2). In this case, Kanto-the most powerful economic area-produces lots of output and shares it with the other areas due to no transfer costs. This is similar to the unified regulation case. In fact, we can show that unified regulation is a preferable strategy if we do not consider the transfer costs and assume the same climate damage ratio in all areas (see Appendix B).

The climate damage ratio would depend on geological characteristics, economic levels, and so forth. On the other hand, although no transfer costs are one of the extreme assumptions, there will be situations close to the assumption if a region is not spatially large or if a region has developed transportation networks or there is no conflict between the areas in a region. Taking a look around the world, Japan might be a region that is relatively relevant in terms of these conditions. Alternatively, for Russia and the United States (both single countries with huge land masses), it may be better to have different regulations for different areas in the country. However, as shown in the comparative analysis of a different regulation case and a unified regulation case, a unified regulation case may produce higher output.

We analyzed three cases for the three scenarios. The different regulations case and the unified regulation case were based on the model without capital transfer between areas, whereas the output redistribution case was based on the model with free capital transfer between areas. Although both models are extreme situations, the regions closer to the former situation possibly have an incentive to use the different regulations policy depending on the emissions target. The regions close to the latter situation would probably prefer unified regulation. However, we should note that these models do not consider industries. In the real world, emission regulation policies are considered to depend largely on condition involving industrial structure and location. Although this is true, we found that regulation policy may change depending on the capital transfer costs or the targets to be achieved. In the future, it will be necessary to clarify how much these factors influence regulation policy decisions when industry is considered.

## Conclusions

We have proposed a model based on the RICE model and have analyzed the impact of global warming using three scenarios. We obtained information on the optimal emission levels in each region by using the RICE model. Our proposed model uses the results and calculates the optimal allocation of the assigned $\text {CO}_2$ emission volume to the subdivided areas within a region.

As a result, we found that impact in the future will differ greatly due to the differences in the implemented scenarios, i.e., *baseline*, *High*, and *Low*. The regulation in the case of *High* will start in 2100 whereas that in the *Low* scenario claims that all areas must stop $\text {CO}_2$ emissions by 2035. However, the more developed areas should implement stricter regulations in all scenarios. By comparing the case of different regulations for each area, the case of a unified regulation for all areas, and the case of output redistribution, we found that different effects will result from the different regulation policies in each scenario.

We analyzed three cases for the three scenarios. Focusing on the different regulation policy and a unified regulation policy, these different policies have an insignificant effect on total output compared with the effect of the different scenarios, but reviewing the policy has potential to change the cost by 5 billion USD per year. In addition, the different regulation policy influences the disparities of the areas and may decrease the total output even if the total utility increases. In the case of the *Baseline*, the total output is large compared with that in the unified regulation case, while there is an increase or decrease during different years. Alternatively, the total output becomes small compared with the unified regulation case, the case of the *Low* scenario.

The different regulations case and the unified regulation case are based on the model without capital transfer between the areas, whereas the output redistribution case is based on the model with free capital transfer between the areas. In all scenarios, the case of output redistribution produces more at all time periods, and the present values of the output are also higher than in the other cases. By comparing these cases, we can found that regulation policy may change depending on the capital transfer costs or the targets to be achieved.

## Data Availability

The datasets used in this article are available upon request.

## Appendix A

### The initial values and the exogenous variables in the model

The TFP, *A*, and the population, *L*, are the exogenous variables which vary from area to area. These quantities take the form

25 $$\begin{aligned} A_{j,t+1}=\, & {} A_{j,t}[1+\zeta _{1,j}\cdot e^{-\zeta _{2,j}\cdot t}] \end{aligned}$$

26 $$\begin{aligned} L_{j,t+1}=\, & {} L_{j,t}\left[ \frac{\zeta _{3,j}}{L_{j,t}} \right] ^{\zeta _{4,j}}. \end{aligned}$$

The level of carbon intensity, $\sigma _{i,t}$, is given by

27 $$\begin{aligned} \sigma _{j,t+1}=\, & {} \sigma _{j,t}\cdot e^{g_{\sigma }(t)} \end{aligned}$$

28 $$\begin{aligned} g_{\sigma }(t+1)=\, & {} 0.995\cdot g_{\sigma }(t) \end{aligned}$$

The parameter of the abatement cost, $\theta ^1_{j,t}$, is denoted

29 $$\begin{aligned} \theta ^1_{j,t}=\frac{ 0.975^{t-1}\cdot \sigma _{j,t}}{5.2} \end{aligned}$$

with $\sigma _{j,t}$. This parameter represents the technological cost, which monotonically decreases with respect to time.

The parameters and the initial values are listed in Table 3. These assumptions are similar to that in Nordhaus [27]. In addition, we use the result from the RICE model to which Nordhaus [27] and Nordhaus and Yang [29] are applied as the climate damage ratio, $\Omega _{0,t}$, and the upper bound of the total $\text {CO}_2$ emission, $E_{0,t}$.

**Table 3:** The initial values and the parameters

	Hokkaido	Tohoku	Kanto	Chubu	Kansai	Chugoku	Shikoku	Kyushu
$A_{j,1}$	5.598	6.619	9.855	8.172	8.061	7.220	6.357	6.295
$L_{j,1}$	5.382	9.006	42.656	21.409	22.500	7.441	3.865	14.432
$Q_{j,1}$	0.159	0.289	1.692	0.799	0.778	0.255	0.121	0.416
$K_{j,1}$	1.362	1.729	4.427	3.387	2.889	1.343	0.783	2.309
$E_{j,1}$	0.078	0.107	0.506	0.287	0.281	0.171	0.053	0.176
$\sigma _{j,1}$	0.485	0.358	0.290	0.348	0.350	0.651	0.426	0.410
$g_{\sigma }(1)$	− 0.0152	− 0.0152	− 0.0152	− 0.0152	− 0.0152	− 0.0152	− 0.0152	− 0.0152
$\zeta _{1,j}$	0.076	0.076	0.076	0.076	0.076	0.076	0.076	0.076
$\zeta _{2,j}$	0.005	0.005	0.005	0.005	0.005	0.005	0.005	0.005
$\zeta _{3,j}$	4.005	6.203	39.252	17.691	18.384	6.063	2.822	11.996
$\zeta _{4,j}$	0.153	0.160	0.082	0.149	0.145	0.150	0.170	0.146

## Appendix B

Here, we consider the problem formulated by Eqs. (17)–(24). We can express Eq. (20) as

30 $$\begin{aligned} Y_{i,t}= {\left\{ \begin{array}{ll} x_{i,t}\sum \nolimits _j^M Q_{j,t} &\quad i=1,\ldots , M-1 \\ \left( 1-\sum \nolimits _r^{M-1} x_{r,t}\right) \sum \nolimits _j^M Q_{j,t} &\quad i=M \end{array}\right. }, \end{aligned}$$

where $x_{i,t}$ is the rate of the output that area *i* accounts for by the redistribution. Then, the Lagrangian function for the problem is

31 $$\begin{aligned} L&= {} \sum _{j}^M\sum _{t}^{T_{max}}(1+\rho )^{-t+1}U_{j,t}\left( Y_{j,t}-I_{j,t}\right) \\&\quad+\sum _{j}^M\sum _{t}^{T_{max}}\lambda ^k_{j,t}\left[ I_{j,t}+\left( 1-\delta \right) K_{j,t}-K_{j,t+1}\right] \nonumber \\&\quad+\sum _{j}^M\sum _{t}^{T_{max}}\lambda ^e_{j,t}\left[ \sum _r^M E_{r,t}-E_{0,t}\right] . \end{aligned}$$

Hence, at a time *t*,

32 $$\begin{aligned} \frac{\partial L}{\partial x_{j,t}} & = (1+\rho )^{-t+1}\frac{\partial U_{j,t}}{\partial Y_{j,t}}\sum _r^M Q_{r,t}\\&\quad-(1+\rho )^{-t+1}\frac{\partial U_{M,t}}{\partial Y_{M,t}}\sum _r^M Q_{r,t}\nonumber \\& = (1+\rho )^{-t+1}\sum _r^M Q_{r,t}\left[ \frac{\partial U_{j,t}}{\partial Y_{j,t}}-\frac{\partial U_{M,t}}{\partial Y_{M,t}}\right] =0 \end{aligned}$$

33 $$\begin{aligned} \frac{\partial L}{\partial \mu _{j,t}} & = (1+\rho )^{-t+1}\sum _r^M\frac{\partial U_{r,t}}{\partial Y_{r,t}}\frac{\partial Y_{r,t}}{\partial \mu _{j,t}}+\sum _r^M\lambda ^e_{r,t}\frac{\partial E_{j,t}}{\partial \mu _{j,t}}\nonumber \\ & = (1+\rho )^{-t+1}X_{j,t}[1-\Omega _{0,t}]\left[ -\frac{\partial \Lambda _{j,t}}{\partial \mu _{j,t}}\right]\\&\quad\times \left( \sum _r^{M-1} \frac{\partial U_{r,t}}{\partial C_{r,t}}x_{r,t}+ \frac{\partial U_{M,t}}{\partial C_{M,t}}\left[ 1-\sum _r^{M-1}x_{r,t} \right] \right) \nonumber \\ & \quad - \sum _r^{M}\lambda ^e_{r,t}X_{j,t}=0, \end{aligned}$$

where $X_{j,t}=\sigma _{j,t}A_{j,t}K_{j,t}^{\gamma }L_{j,t}^{1-\gamma }$. Then, from Eq. (32), we obtain

34 $$\begin{aligned} \frac{\partial U_{j,t}}{\partial Y_{j,t}}=\frac{\partial U_{M,t}}{\partial Y_{M,t}}, j=1,\cdots , M-1. \end{aligned}$$

By substituting Eq. (34) into Eq. (33) and eliminating $\sum _r^{M}\lambda ^e_{r,t}$, we can get

35 $$\begin{aligned} \frac{1-\Omega _{0,t}}{\sigma _{i,t}}\left[ -\frac{\partial \Lambda _{j,t}}{\partial \mu _{i,t}}\right] =\frac{1-\Omega _{0,t}}{\sigma _{j,t}}\left[ -\frac{\partial \Lambda _{j,t}}{\partial \mu _{j,t}}\right] , i,j\in {\mathbb {M}} \end{aligned}$$

From Eqs. (22) and (29),

36 $$\begin{aligned} \frac{\partial \Lambda }{\partial \mu _{j,t}}=\frac{ 0.975^{t-1}\cdot \sigma _{j,t}}{5.2}\theta ^2\mu _{j,t}^{\theta ^2-1}. \end{aligned}$$

Since,

37 $$\begin{aligned} \mu _{i,t}=\mu _{j,t}, i,j\in {\mathbb {M}}. \end{aligned}$$

## Abbreviations

GHG: greenhouse gas
RICE model: the regional integrated climate-economy model
IAMs: integrated assessment models
DICE model: the dynamic integrated climate-economy model
TFP: total factor productivity
CAO: Cabinet office, Government of Japan
IPSS: National Institute of Population and Social Security Research
RCP: Representative Concentration Pathways

## Footnotes

1 Tokyo Metropolitan Government.

2 The abatement cost is set to decrease with time in this model because of technological innovation (see Appendix A).

3 This study uses 2005 prices as the standard.
